# Mapping Quantitative Trait Loci Controlling High Iron and Zinc Content in Self and Open Pollinated Grains of Pearl Millet [*Pennisetum glaucum* (L.) R. Br.]

**DOI:** 10.3389/fpls.2016.01636

**Published:** 2016-11-23

**Authors:** Sushil Kumar, Charles T. Hash, Nepolean Thirunavukkarasu, Govind Singh, Vengaldas Rajaram, Abhishek Rathore, Senthilvel Senapathy, Mahesh D. Mahendrakar, Rattan S. Yadav, Rakesh K. Srivastava

**Affiliations:** ^1^Plant Biotechnology Centre, Swami Keshwanand Rajasthan Agricultural UniversityBikaner, India; ^2^International Crops Research Institute for the Semi-Arid TropicsPatancheru, India; ^3^Centre of Excellence in Agricultural Biotechnology, Anand Agricultural UniversityAnand, India; ^4^International Crops Research Institute for the Semi-Arid TropicsNiamey, Niger; ^5^Indian Agricultural Research Institute, New DelhiIndia; ^6^Directorate of Oilseeds ResearchHyderabad, India; ^7^Crop Genetics, Genomics and Breeding Division, Aberystwyth UniversityAberystwyth, UK

**Keywords:** RIL, QTL, iron and zinc, biofortification, pearl millet

## Abstract

Pearl millet is a multipurpose grain/fodder crop of the semi-arid tropics, feeding many of the world’s poorest and most undernourished people. Genetic variation among adapted pearl millet inbreds and hybrids suggests it will be possible to improve grain micronutrient concentrations by selective breeding. Using 305 loci, a linkage map was constructed to map QTLs for grain iron [Fe] and zinc [Zn] using replicated samples of 106 pearl millet RILs (F_6_) derived from ICMB 841-P3 × 863B-P2. The grains of the RIL population were evaluated for Fe and Zn content using atomic absorption spectrophotometer. Grain mineral concentrations ranged from 28.4 to 124.0 ppm for Fe and 28.7 to 119.8 ppm for Zn. Similarly, grain Fe and Zn in open pollinated seeds ranged between 22.4–77.4 and 21.9–73.7 ppm, respectively. Mapping with 305 (96 SSRs; 208 DArT) markers detected seven linkage groups covering 1749 cM (Haldane) with an average intermarker distance of 5.73 cM. On the basis of two environment phenotypic data, two co-localized QTLs for Fe and Zn content on linkage group (LG) 3 were identified by composite interval mapping (CIM). Fe QTL explained 19% phenotypic variation, whereas the Zn QTL explained 36% phenotypic variation. Likewise for open pollinated seeds, the QTL analysis led to the identification of two QTLs for grain Fe content on LG3 and 5, and two QTLs for grain Zn content on LG3 and 7. The total phenotypic variance for Fe and Zn QTLs in open pollinated seeds was 16 and 42%, respectively. Analysis of QTL × QTL and QTL × QTL × environment interactions indicated no major epistasis.

## Introduction

Billions of people worldwide, especially in developing countries, suffer from the sinister form of hunger called micronutrient malnutrition or hidden hunger, a particular form of unnoticed undernutrition. Micronutrient deficiencies can exist in populations even where the food supply is adequate in terms of meeting energy requirements. Humans need more than 22 mineral elements; some of them are required in large amounts, but others, such as Fe, Zn, Cu, I, and Se, are required in trace quantities (Grusak and Cakmak, 2005).

Micronutrient malnutrition resulting from the dietary deficiency of critically important minerals such as iron (Fe) and zinc (Zn) has been reported to be a major food-related primary health problem among populations of the developing world—including those in India—that are heavily dependent on cereal and legume-based diets and have limited access to meat, fruits and vegetables (Sandstead, 1991; Gibson, 1994; Welch and Graham, 2002). Worldwide, one out of seven people suffers from hunger; and most of them are poor people, particularly women, infants, and children. Micronutrient malnutrition persists as a major problem that not only affects vital growth in children but also actively damages the cognitive development of children, resulting in blindness, lowering disease resistance and reducing the likelihood that mothers survive childbirth (Sharma et al., 2003).

To address the occurrence of mineral deficiencies in human populations, plant scientists are devising methods of applying fertilizers and/or using plant breeding strategies to increase the concentrations and/or bioavailability of mineral elements in agricultural produce (Cakmak et al., 2004; Graham et al., 2007; Pfeiffer and McClafferty, 2007; Cakmak, 2008; White and Broadley, 2009). These approaches are termed ‘agronomic’ and ‘genetic’ biofortification, respectively. The biofortification approach involves a set of one-time, fixed costs in developing breeding methodologies, breeding nutritional quality traits into current crop varieties, and adapting these varieties to diverse environments. Breeding for higher trace mineral content in the consumed plant parts may not incur a yield penalty (Graham and Welch, 1996; Graham et al., 2007). It is known that biofortified crops can produce nutrient dense grains even in relatively poor soils with target nutrients within the critical range (Kanatti et al., 2014; Valentinuzzi et al., 2015).

Pearl millet [*Pennisetum glaucum* (L.) R. Br.] is a highly nutritious cereal with high levels of metabolizable energy, protein and micronutrients, a more balanced amino acid profile than wheat and rice and may other cereals, and low glycemic index (Singh et al., 1999; Sehgal et al., 2004). It is an important and low-cost source of food for the poor in West Africa and India. Its contribution of micronutrients, especially iron [Fe] and zinc [Zn], is higher varying from 30 to 50% of the intake of these micronutrients from cereals (Rao et al., 2006). An accelerated incorporation of the gene(s)/QTLs that enhance the nutrient content in staple crops will largely determine the success of various nutritional breeding schemes (Bohra et al., 2016). This study was undertaken with a hypothesis that measurable genetic variation exists in pearl millet, and such variation could be utilized for mining and mapping of QTLs for grain Fe and Zn content. In this study, for the first time, we report linkage relationship and phenotypic effects for grain Fe and Zn content QTLs in pearl millet.

## Materials and Methods

### Mapping Population

Recombinant inbred line (RIL) population advanced by single seed descent method from F_2_ to F_6_ was used in the present study. The population was derived from an intraspecific cross: ICMB 841-P3 × 863B-P2; comprised 144 lines, and was segregating for grain [Fe] and [Zn] contents, respectively. The parent ICMB 841 was bred by pure-line selection for downy mildew resistance within an outcrossed seed lot of elite seed parent maintainer line MS 5141B. ICMB 841 is the seed parent maintainer line of several popular dual-purpose hybrids released in India (Singh, 1990; Govila et al., 1997). Inbred maintainer line 863B was bred by pure-line selection at ICRISAT-Patancheru within a sample of Iniadi landrace material from Togo in West Africa (Rai et al., 2008). Compared to ICMB 841, 863B has larger grain size (and associated broader leaf blades, thicker stems, and thicker panicles), and better drought tolerance, downy mildew resistance, and stover quality. It was originally selected as a parent for mapping population development based on its combination of agronomic eliteness and superior combining ability for grain filling under terminal drought stress (Yadav et al., 2004; Bidinger et al., 2007). Standard agronomic practices and need-based plant protection measures were adopted uniformly to raise the crop.

### Experimental Conditions

The experiment was conducted in an alpha-lattice design on Alfisol fields at ICRISAT-Patancheru (17.51°N; 78.27°E) with two replications in two environments during 2009 late *Kharif* (E1) and 2010 Summer (E2). Randomization was performed using IRRISTAT 5.0. The trials were conducted with a total of 120 entries (106 RILs + 2 parents + 4 checks). Each entry was sown in two-row plots (length 4 m) with 60 cm inter- and 15 cm intra-row spacing to produce bulks of selfed seeds as well as open-pollinated seeds for mineral element analysis. On the basis of seed setting, 15–20 panicles were harvested manually to produce the self-bulk seed samples while 5–10 panicles were harvested to produce the open-pollinated bulk seed samples, from each plot.

### Mineral Analysis

Well dried F_6_ panicles (Self-and open-pollinated) were threshed using a thresher (Winter Steiger, Germany) to avoid any metal contamination. Briefly, cleaned grains were dried in hot air oven at 80°C for 24 h followed by grinding of 10 g of whole grain using a cyclone sample mill (Udy Corporation, USA). The grain powder (1 g) was digested in fume-hood with 10 ml triacid mixture of 5:2:1 by volume of concentrated nitric acid, sulphuric acid, and perchloric acid until clear white residue was obtained. After overnight cold digestion, the samples were digested at 120°C for 1 h, followed 2-h digestion at 230°C temperature. Required volume (75 ml) was made after completion of the digestion process, and digests were analyzed using an atomic absorption spectrophotometer (Model: SpectraAA 20, Varian, USA) according to the (Velu et al., 2006). Each sample was measured thrice and the means of these triplicate observations were used to represent the sample’s mineral elements contents. The mean observation for each sample was converted into the amount of mineral elements (mg kg^-1^) in each sample using Microsoft Excel. Grain Fe and Zn contents were computed as parts per million (ppm). Trait correlations between iron and zinc contents were determined using SAS software.

### Genotyping of Mapping Population

DNA was extracted following Mace et al. (2003) and was normalized to 5–10 ng μL^-1^ concentrations for SSR, whereas, for DArT marker-based genotyping, DNA was diluted to 50 ng μL^-1^. All the RILs and their two parents were genotyped by both simple sequence repeat (SSR) and DArT markers. For SSR analysis, microsatellite markers from different sources [EST-SSRs (*Xicmp* and *Xipes* series), gSSRs (*Xpsmp* and *Xctm* series), and STS (*Xpsmp(sts)*] were used to identify polymorphism between ICMB 841-P3 and 863B-P2 (Allouis et al., 2001; Qi et al., 2001, 2004; Budak et al., 2003; Senthilvel et al., 2008; Rajaram et al., 2013). The forward primers of *Xipes* series SSRs were synthesized with an m13-sequence (5′CACGACGTTGTAAAACGAC3′) tail on the 5′ end. SSRs were amplified in a 10 μl PCR reaction mixture containing 10–15 ng of genomic DNA, 2 pmol of each primer, 2 mM MgCl2, 0.4 mM of each dNTP, 1× reaction buffer, and 0.2 U Taq polymerase (Bioline). PCR conditions were as follows: denaturation at 94°C for 5 min, followed by 10 cycles of denaturation at 94°C for 15 s, annealing at 61°C to 51°C (touch-down cycles) for 30 s, and extension at 72°C for 30 s, followed by 40 cycles of denaturation at 94°C for 10 s, annealing at 54°C for 30 s, and extension at 72°C for 30 s, followed by final extension at 72°C for 20 min. PCR amplification was checked on 1.2% agarose gels and PCR products were separated by capillary electrophoresis on an ABI3730xl sequencer and their sizes were determined using GeneMapper v4.0 software (Applied Biosystems, USA).

For DArT genotyping, DNA samples (50 ng μL^-1^) was submitted to the Genotyping Service Laboratory (GSL) at ICRISAT-Patancheru, where RIL population was genotyped using an array of 6912 DArT clones developed by *Pst*I/*Bam*II complexity reduction. DArTsoft, a software package developed at DArT P/L, was used to automatically analyze the output data. The DArTsoft-generated 0–1 scores of the polymorphic DArT markers detected among the inbred lines and parents of the particular RIL population were converted to A–B scores and used as input for mapping.

To construct the map, 38 RILs (26% of the population) were discarded from the original set of 144 lines because they were contaminated or had loci with non-parental alleles. Further, we used only the remaining 106 RILs for mapping of the DArT and EST-SSR markers (*Xipes* series).

### Linkage Map Construction

A standard χ^2^-test was employed to test the segregation at each marker locus for deviation from the expected Mendelian segregation. Deviations of marker loci from the expected proportion of heterozygous alleles were tested by a *t*-test. Residual heterozygosity was considered while mapping. Linkage analysis was performed by using the MAPMAKER/EXP 3.0 program (Lander et al., 1987). The critical logarithm of odds (LOD) score for the test of independence of marker pairs was set at 3.0 and maximum recombination fraction (𝜃) of 0.49. In a preliminary analysis of the data, markers were deleted that had high segregation distortion (*P* ≤ 0.01). Subsequently, the linkage analysis of genotyping data for all the markers including those with distorted segregation was used for linkage analysis. Further, the RECORD software (Van Os et al., 2005) was used to get the most likely marker order in each linkage group. The order of markers in each linkage group was finalized by RECORD software (Van Os et al., 2005), and distances between markers loci calculated using the Haldane mapping function. A graphical representation of the map was drawn using MapChart software (Voorrips, 2002). The consensus maps developed by Rajaram et al. (2010), and DArT marker-saturated linkage maps developed by Supriya et al. (2011) were considered as a reference map for grouping of the markers and to construct the map in the present study.

### Trait Analysis

The phenotypic variance was partitioned using the residual maximum likelihood (ReML) algorithm with a mixed model, where replication and block were considered to be fixed effects, while genotypes were random effects, to obtain the best linear unbiased predictions (BLUPs) of the performance of genotypes for each observed trait (Patterson and Thompson, 1971). Pearson correlation coefficients were carried out for studied traits; namely, Fe and Zn content (ppm) using PROC CORR in SAS (SAS Institute Inc., 1999). Because a normal distribution could not be assumed for all observed variables, Spearman’s rank correlation (*r*_s_) was also used as a robust estimation of the correlation coefficient. Similarly, genotypic correlations were also estimated. The significance of the correlation coefficients at *P* ≤ 0.05 and 0.01 was indicated as ^∗^ and ^∗∗^, respectively. Broad-sense heritability on plot means basis was computed from the estimates of genetic (σ_g_^2^ ) and residual (σ_e_^2^ ) variances using progeny means across RILs in each environment for both traits using PROC MIXED in SAS (SAS Institute Inc., 1999). Heritability (*H*^2^) was estimated as explained by Falconer (1989).

### QTL Detection

Composite interval mapping (CIM) (Zeng, 1994) was used to search for QTL using the BLUP data of each trait. CIM was performed in PLABQTL (Utz and Melchinger, 1996) on an RIL population of 106 lines with 2 cM increments. To declare a putative QTL as statistically significant, a minimum LOD score of 3 was fixed according to the Bonferroni correction. The critical LOD threshold was analyzed empirically for each trait using 1,000 permutation runs. The proportion of the phenotypic variance explained by the QTL was determined by the estimator R2adj. The putative QTLs detected for each of the respective traits were assigned to linkage groups based on the map positions of their flanking markers. QQE interactions were identified using QTLNetwork 2.0.

## Results

### Performance of the Population

The average performance and the descriptive statistics for traits in two parents and RILs grown in 2009 and 2010 are given in **Table 1**. The parental BLUPs differences in Zn_OP was non-significant in E1 but found significant in E2 and in the joint dataset analysis. The mean BLUPs of [Fe] in self-seeds were higher in E1 for both parents. Selfed seed [Zn] BLUPs of ICMB 841-P3 were the same in both screening environments, whereas 863B-P2 had higher selfed seed [Zn] in E2. [Fe] and [Zn] in OP seeds of ICMB 841-P3 were higher in E1; while in the case of 863B-P2, [Zn] was greater in E2 and uniform [Fe] in OP seeds was observed in the two screening environments.

**Table 1:** Descriptive statistics of phenotypic values observed in RILs derived from cross (ICMB 841-P3 × 863B-P2), and their parental lines, in two different environments at ICRISAT-Patancheru, and across these two environments.

Trait	Environment	ICMB 841 (P1)	863B (P2)	RILs		P1 vs. P2	P1 vs. RILs	P2 vs. RILs
		Mean	Mean	Mean	Range	Pr > F	Pr > F	Pr > F
Fe_Self	2009	44.59 ± 3.56	97.23 ± 3.51	64.43 ± 0.61	28.4–116.5	^∗∗^	^∗∗^	^∗∗^
	2010	41.12 ± 3.54	93.16 ± 3.53	72.50 ± 0.60	33.8–124.0	^∗∗^	^∗∗^	^∗∗^
	Pooled	44.01 ± 5.78	92.93 ± 5.77	68.48 ± 3.88	28.4–124.0	^∗∗^	^∗∗^	^∗∗^
Zn_Self	2009	47.72 ± 2.96	62.08 ± 2.92	59.34 ± 0.51	28.7–101.9	^∗∗^	^∗∗^	ns
	2010	47.40 ± 2.85	69.30 ± 2.84	69.19 ± 0.48	33.8–119.8	^∗∗^	^∗∗^	ns
	Pooled	49.15 ± 6.26	64.79 ± 6.26	64.21 ± 4.73	28.7–119.8	^∗∗^	^∗∗^	ns
Fe_OP	2009	39.39 ± 2.49	60.91 ± 2.35	48.36 ± 0.41	25.3–77.4	^∗∗^	^∗∗^	^∗∗^
	2010	28.70 ± 1.59	60.29 ± 1.68	43.08 ± 0.26	22.4–69.3	^∗∗^	^∗∗^	^∗∗^
	Pooled	34.23 ± 3.33	60.56 ± 3.33	45.68 ± 2.61	22.4–77.4	^∗∗^	^∗∗^	^∗∗^
Zn_OP	2009	44.89 ± 1.98	45.59 ± 1.96	45.77 ± 0.34	21.9–73.7	ns	ns	ns
	2010	33.49 ± 1.42	48.62 ± 1.51	45.73 ± 0.24	24.5–67.3	^∗∗^	^∗∗^	ns
	Pooled	40.04 ± 2.65	46.97 ± 2.66	45.72 ± 1.11	21.9–73.7	^∗^	^∗^	ns

*Fe _Self, self-pollinated grain Fe content (ppm); Zn_Self, self-pollinated grain Zn content (ppm); Fe_OP, open-pollinated grain Fe content (ppm); Zn_OP = open-pollinated grain Zn content (ppm). ^∗^Significant at 5% level; ^∗∗^significant at 1%level.*

The performance of RILs for traits studied also varied in the two screening environments. The BLUPs means of RILs for selfed seed [Fe] and [Zn] were higher in E2, while RIL BLUPs means for Fe_OP and Zn_OP were higher in E1. The differences in selfed seed [Zn] mean BLUPs values of ICMB 841-P3 and RILs was non-significant in E1. On the basis of pooled environment analyses, the mean performances of RILs for [Zn] (selfed as well as OP seeds) were non-significantly different from 863B-P2 across the two screening environments. The differences in BLUPs means of RILs and 863B-P2 were significant only for [Fe] (in self and OP seeds) in the joint analysis of these two datasets. The BLUPs means of both parents and RILs for [Fe] and [Zn] in selfed seeds were higher than those of [Fe] and [Zn] in OP seeds in three datasets.

### Variance Components

The genotypic variances for [Fe] of selfed and OP seeds, and [Zn] of OP seed were higher in E1, while the [Zn] of self-seeds exhibited higher genotypic variance in E2 (**Table 2**). Considering the analysis across the two screening environments, the results showed that variances due to genotypes were significant (at *P* < 0.01) for all observed traits. Likewise, variance due to G × E interactions were significant (at *P* < 0.01) for all observed traits across these two environments except for Fe_OP, which was significant at *P* < 0.05. In general, however, genetic variances were substantially larger (and often an order of magnitude larger) than those for G × E interactions, for the observed traits in this RIL population. Numerically higher genetic variances were detected for [Fe] and [Zn] of selfed seed in E2 than E1. For [Fe] and [Zn] of OP seeds, the genetic variances were numerically, but not significantly, greater in E1 than E2.

**Table 2:** Genotypic variance (σ^2^g), G × E interaction (σ^2^g × E), standard error (SE) and operational heritability (broad-sense; H^2^) for traits observed in the (ICMB 841-P3 × 863B-P2)-derived RIL population, in two different environments at ICRISAT-Patancheru, and across these two environments.

Trait	E1			E2			Pooled				
	σ^2^g	*SE*	*H*^2^	σ^2^g	*SE*	*H*^2^	σ^2^g	*SE*	σ^2^g × E	*SE*	*H*^2^
Fe_Self	291.33	45.94	0.74	283.28	44.19	0.74	260.00	40.71	28.47	8.94	0.66
Zn_Self	173.51	27.69	0.71	224.51	34.58	0.78	162.72	26.57	30.19	7.79	0.62
Fe_OP	72.81	12.63	0.61	60.73	9.32	0.75	60.81	9.61	5.09	2.42	0.61
Zn_OP	42.45	7.65	0.56	38.48	6.12	0.70	28.61	5.52	11.70	2.87	0.44

### Heritability and Correlation Analysis

All observed traits, except Zn_OP in E1 (late *Kharif* 2009), were very highly heritable (>0.60) as per the scale of Robinson et al. (1949), having *H*^2^ values more than 0.60. However, partitioning out the genotype by environment interaction component of variance reduced the *H*^2^ values for the combined dataset across 2009 and 2010 (**Table 1**). The operational heritability for [Fe] in self-seeds was 0.74 in both the environments while for [Fe] in OP seeds, *H*^2^ (0.75) in 2010 was higher than for [Fe] in self-seeds. The variation in the two environments slightly lowered the estimates for operational heritabilities across these two environments.

A very strong significantly positive association was detected between [Fe] and [Zn], while correlations were moderate for trait pairs [Fe] and Fe_OP, [Fe] and Zn_OP, Fe_OP and Zn_OP, [Zn] and Zn_OP, and [Zn] and Fe_OP (**Table 3**). Correlation coefficients at the genotypic level were higher than those detected at a phenotypic level for all observed characters. The Genotypic correlation was found more significant than phenotypic correlation indicating that there was the prevalence of environmental interaction with genotype on the observed traits. Similar to phenotypic correlations, the genotypic correlations in all three datasets was very strong and significantly positive between trait pairs [Fe] and [Zn], [Fe] and Fe_OP, [Fe] and Zn_OP, [Zn] and Zn_OP, Fe_OP and Zn_OP, and [Zn] and Fe_OP.

**Table 3:** Genotypic correlation coefficients between traits in RIL population derived from (ICMB 841-P3 × 863B-P2).

Trait	Environment	Fe	Zn	Fe_OP	Zn_OP
Fe	1	1			
Fe	2	1			
Fe	Pooled	1			
Zn	1	0.891^∗∗^	1		
Zn	2	0.877^∗∗^	1		
Zn	Pooled	0.885^∗∗^	1		
Fe_OP	1	0.915^∗∗^	0.771^∗∗^	1	
Fe_OP	2	0.843^∗∗^	0.594^∗∗^	1	
Fe_OP	Pooled	0.873^∗∗^	0.666^∗∗^	1	
Zn_OP	1	0.696^∗∗^	0.845^∗∗^	0.684^∗∗^	1
Zn_OP	2	0.777^∗∗^	0.769^∗∗^	0.784^∗∗^	1
Zn_OP	Pooled	0.768^∗∗^	0.816^∗∗^	0.767^∗∗^	1

*^∗∗^Significant at 1% level.*

### Molecular Analysis

#### Parental Polymorphism

A total of 342 SSR primer pairs (IPES, PSMP, CTM, and ICMP series) were used for polymorphism survey of both parental lines. Of them, 124 (36.2%) SSRs detected polymorphism between the two parents. Polymorphic SSR markers were detected by 65 *Xipes*, 36 *Xpsmp*, 16 *Xicmp* and 5 *Xctm* series primer pairs and 2 STS primer pairs. A set of 279 (4.04%) polymorphic DArT clones were identified on the array for ICMB 841 and 863B.

#### Linkage Map Construction

Out of 124 polymorphic SSR markers (36 *Xpsmp*, 16 *Xicmp*, 65 *Xipes*, 5 *Xctm* and 2 STS), we could map only 25 *Xpsmp*, 9 *Xicmp*, 58 *Xipes*, 3 *Xctm* and 2 STS markers on 106 selected RILs out of 144. Rest of the polymorphic markers could not be mapped due to certain problems like dominant inheritance, lack of linkage, very high percentage of segregation distortion toward one parent. Likewise, 208 (74.55%), out of 279, polymorphic DArT markers were used to construct the map as the remaining were unlinked. In all, out of the 403 markers genotyped, 305 (75.6%) markers were used for linkage map construction markers with a LOD score 3.0. These assigned markers were 208 DArTs, 95 SSRs and 2 STSs (**Table 4**).

**Table 4:** Linkage group wise summary of the distorted markers in (ICMB 841-P3 × 863B-P2) genetic linkage map.

LG	SSR	Skewed SSR	DArT	Skewed DArT	Total markers	Total Skewed	Skewed %	Total distance	Average distance
1	16	3	49	23	65	26	40	374.8	5.76
2	17 (16 + 1 STS)	16	20	19	37	35	95	264.3	7.14
3	8	8	24	17	32	25	78	212.6	6.64
4	11 (10 + 1 STS)	2	46	23	57	25	44	192.8	3.38
5	11	0	19	12	29	12	41	192	6.62
6A	1	1	9	3	10	4	40	18.8	1.88
6B	13	0	18	13	32	14	44	94.2	2.94
6C	5	2	2	2	7	4	57	104.8	14.9
7	15	10	21	13	36	23	64	294.4	8.17
Total	97	43	208	125	305	168	55	1748.7	5.73

**FIGURE 1:**
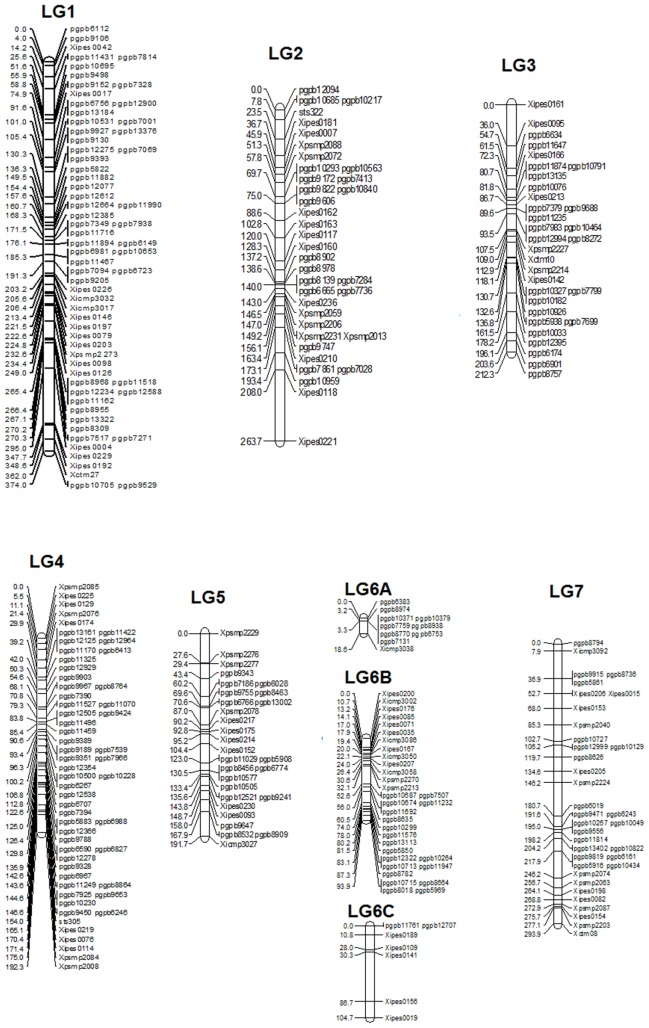
**Linkage Map of (ICMB 841-P3 × 863B-P2)**.

The linkage map had a total map length of 1748.7 cM (Haldane). The average interval size was 5.73 cM. Taking into consideration of the genome size of pearl millet (2450 Mbp) the current spans 1401 kb per cM. The individual LGs ranged from 374.8 cM for LG1 with the highest number of markers (65) to 18.8 cM for LG6A with 10 markers, while the least number of markers were assigned on LG6C (7) followed by LG5 (29). The average linkage group length was 249.8 cM with an average of 43.5 loci. The average adjacent-marker interval lengths ranged from 1.88 cM (LG6A) to 14.9 cM (LG6C) followed by 8.17 cM (LG7). Map distance between adjacent markers varied from 0 to 56.4 cM (LG6B) and 20.4% of the intervals (63 out of 305 intervals) were greater than 10 cM. Ten map-intervals were observed with markers separated more than 25 cM on LG1 (3), LG2 (1), LG3 (1), LG5 (1), L6C (1) and LG7 (3) (**Figure 1**)

#### Segregation Distortion

In the present study, a big portion of the distorted markers (119) was skewed toward female parent (ICMB 841) alleles. Maximum regions of distorted segregation were detected on LGs 2 (95%) and 3 (78%). Out of 55% distorted markers, the highest proportion was observed in the DArT markers (75%) as compared to SSRs (25%) (**Table 4**). In summary, out of 305 mapped markers, segregation of 43 SSR and 125 DArT markers were distorted and of these distributions of only 44 were skewed toward 863B.

#### QTL Analysis

##### Fe QTL

**FIGURE 2:**
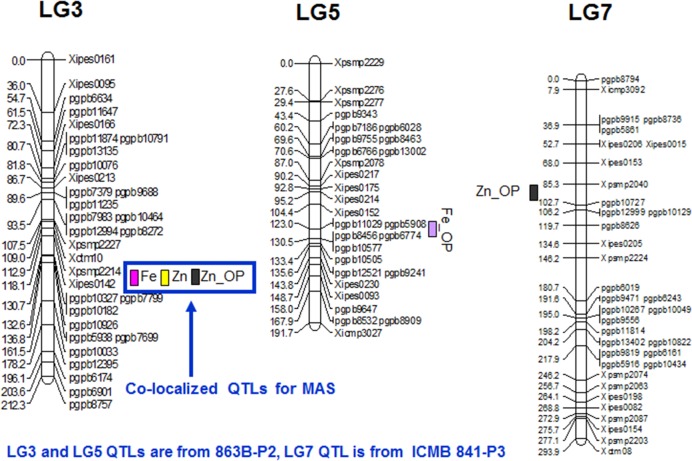
**QTL positions for grain Fe and Zn content as detected by PlabQTL and QTLNetwork software in (ICMB 841-P3 × 863B-P2)- based RIL population**.

In E1, 2 QTLs on LG3 and LG5, with individual partial *R*^2^ values of 13.6% and 10.0%, respectively, were identified for selfed seed iron content, and alleles from parent 863B were favorable at both loci. The adjusted *R*^2^ in E1 for the 2-QTL combined model was 23.9%, which was similar to that for the 5-QTL combined model in E2 (26.3%). The partial *R*^2^ values ranged from 0.1 to 10.0% in E2 and were low compared to E1 values. The QTL on LG3 was common in both environments, with adjusted additive effects of 4.4–6.5 ppm, while the remaining QTLs were environment specific (**Table 5**; **Figure 2**).

**Table 5:** Positions and descriptions of QTLs affecting iron and zinc contents in the (ICMB 841-P3 × 863B-P2)-derived RIL population across the two screening environments (E1 and E2) at ICRISAT-Patancheru.

Trait	QTL Position^$^	Marker Interval	Support Interval	LOD	Partial *R*^2^ (%)	Adjusted additive effects	QEI	*R*^2^ (%)	Additive effects
Fe	3/110^#^	*Xpsmp2214-Xipes142*	106–116	4.68	20.5	8.3	^∗∗^	19.4	4.5
	
	Final simultaneous fit:			LOD = 5.04	Adjusted *R*^2^= 18.9%				
					Adjusted genotypic variation explained = 28.6%				

Zn	3/110^#^	*Xpsmp2214-Xipes142*	106–117	9.66	32.3	8.5	ns	35.9	6.8
	
	Final simultaneous fit:			LOD = 8.54	Adjusted *R*^2^= 30.9%				
					Adjusted genotypic variation explained = 49.8%				

Fe_OP	2/30	*Xpsmp322-Xipes181*	10–38	4.34	0.6	0.6	^∗∗^	18.1	0.7
	5/118	*pgpb11029-pgpb8456*	94–124	4.39	14.6	2.9	ns	18.3	2.6
	
	Final simultaneous fit:			LOD = 4.94	Adjusted *R*^2^= 16.1%				
					Adjusted genotypic variation explained = 26.4%				

Zn_OP	3/110^#^	*Xpsmp2214-Xipes142*	106–116	14.96	34.1	3.7	ns	50.1	3.7
	7/96	*Xpsmp2040-pgpb10727*	90–98	4.77	7.0	-1.2	^∗∗^	19.7	-1.8
	
	Final simultaneous fit:			LOD = 12.84	Adjusted *R*^2^= 41.7%				
					Adjusted genotypic variation explained = 94.8%				

*^$^Leading number: Linkage group; Trailing number: QTL position in cM. ^#^Co-mapped loci. E1, Late Kharif 2009; E2, Summer 2010. ^∗∗^ Significant at 1% level.*

Only one QTL, detected in both environments on LG3, was identified in the across-environment analyses. The QTL-ANOVA using PlabQTL showed significant QEI, with adjusted *R*^2^ = 18.9% and adjusted additive effect = 8.3 ppm. This same single QTL was also detected by QTLNetwork (**Table 6**) but this analysis failed to detect significant QEI.

**Table 6:** Details of the QTLs detected using QTLNetwork and data from the RIL population derived from cross (ICMB 841-P3 × 863B-P2).

Trait	LG/Position^$^	Flanking markers	Support interval	Additive effects^#^	AE1^∗^	AE2
Fe	3/110	*Xpsmp2214-Xipes0142*	105–128	-7.4	–	–
Zn	3/110	*Xpsmp2214-Xipes0142*	107–113	-6.8	–	–
	6B/50	*pgpb10674-pgpb8635*	50–50	3.4	–	–
Fe_OP	3/140	*pgpb5938-pgpb10033*	128–150	-2.7	–	–
Zn_OP	3/108	*Xctm10-Xpsmp2214*	105–114	-3.0	–	–

*^$^Leading number: Linkage group; Trailing number: QTL position in cM. ^#^Additive effects: a negative value indicates that the allele from 863B increases the trait mean, a positive value indicates that the allele from 863B reduces the trait mean. ^∗^AE1, additive effects in test environment 1 (late Kharif 2009) when Q × E effects are significant; AE2, additive effects in test environment 2 (Summer 2010) when Q × E effects are significant; “–”, No QE interaction.*

##### Zn QTL

In E1, 8 putative QTLs influencing the Zn content in selfed seed were mapped, with collective adjusted *R*^2^ of 47.2%, and with 0.01 and 21.8% were their minimum and maximum partial *R*^2^ values. The upper limit of adjusted additive effects in E1 was -6.11 ppm, with ICMB 841B providing the favorable allele. A single QTL on LG3 with partial *R*^2^ of 22.7% was identified in E2. Hence, except for this QTL on LG3, remaining QTLs detected for selfed seed Zn content in E1 were environment specific (**Table 5**; **Figure 2**).

Similar to Fe, only one QTL for selfed seed Zn content was revealed by the PlabQTL across-environment analysis. This QTL at position 110 cM on LG3 had its favorable allele from 863B, with an adjusted *R*^2^ value of 30.9% and an adjusted additive effect of 8.5 ppm. In contrast, QTLNetwork detected two QTLs for selfed seed Zn content using the across-environment dataset. The second QTL detected by QTLNetwork was at position 50 cM on LG6B (**Table 6**), with parent ICMB 841B contributing the favorable allele. QEI was non-significant for QTLs influencing the Zn content of selfed seeds.

##### Fe_OP QTL

Four and two putative QTLs for Fe_OP (Fe content of open-pollinated grains) were detected using the E1 and E2 datasets, respectively. The partial *R*^2^ values in E1 ranged from 1.6 to 18.9%. In E1, the four-QTL model had an adjusted *R*^2^ of 25.9%, with individual QTL adjusted additive effects ranging from 1.0 to 3.0 ppm. In E2, the partial *R*^2^ values for the QTLs detected on LG4 and LG5 were 2.9 and 11.7%, respectively. The two QTLs detected in E2 had a combined adjusted *R*^2^ value of 12.9%, while the adjusted additive effects for QTLs on LG4 and LG5 were 1.0 and 2.2 ppm, respectively. Like TGM_OP, none of the putative QTLs detected for FE_OP were common in the two environments and making them all environment-specific (**Table 5**).

Of the two detected QTLs in joint analysis of these two datasets, one was also detected in E2 while another one was novel. The adjusted *R*^2^ for this 2-QTL genetic model was 16.1% and 863B contributed favorable alleles for both QTLs. QTLNetwork analysis of this combined dataset detected one genomic region at position 140 cM on LG3 that contributed to the control of Fe_OP that was not detected by PlabQTL (**Table 7**). The favorable allele for this QTL was contributed by parent 863B. The QTL-ANOVA showed significant QEI for this trait, but only one QTL on LG2 was significantly influenced by environment.

**Table 7:** Epistatic interactions (additive × additive) detected using PlabQTL for QTL detected in cross (ICMB 841-P3 × 863B-P2).

Trait	Environment	QTL^$^	AA Effect^$^	*R*^2^ [%]	Epistatic effect
Zn	1	1/170	7/176	10.2	-
Zn	1	2/252	3/90	7.1	+
Zn	1	2/252	3/110	6.1	-
Zn	1	3/110	5/82	7.7	+
Zn	1	5/30	7/176	5.9	-
Zn	1	5/82	7/176	7.0	+
Zn_OP	2	3/114	4/14	7.1	-
Fe	2	1/332	5/80	4.9	-
Fe	2	3/110	7/60	10.4	-
	

*^$^Leading number: chromosome; trailing number: position in [cM].*

##### Zn_OP

For Zn_OP, no QTL was found in E1 at significance threshold LOD value 4.2. In contrast, QTL analysis of the E2 dataset revealed the presence of 5 putative QTLs for Zn_OP. The final simultaneous fit analysis yielded an adjusted *R*^2^ value of 32.1% for these five QTLs. Their partial *R*^2^ values ranged from 0.01 to 21.6%. A QTL detected on LG3 in across-season data analyses using both software packages was also detected in E2 (**Tables 6** and **7**), while the remaining four QTLs detected with the E2 dataset were environment-specific (**Table 5**; **Figure 2**). The favorable allele of the QTL on LG3 was inherited from 863B, with an adjusted additive effect of 3.0–3.7 ppm, while the favorable allele for a second QTL detected by PlabQTL was contributed by ICMB 841, with an adjusted additive effect of -1.72 ppm.

#### Epistasis

**FIGURE 3:**
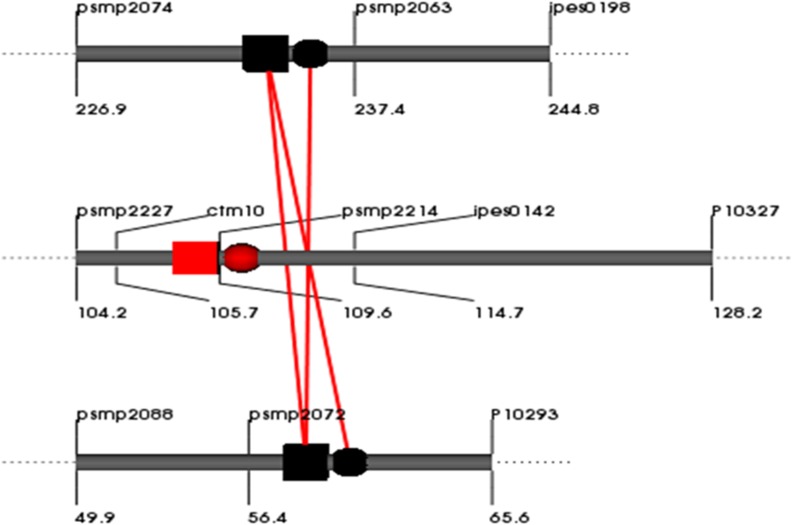
**QQ interaction for grain [Fe] detected using QTLNetwork in and across-environment data from the (ICMB 841-P3 × 863B-P2)- based RIL population.** Red square and circle represent QTL with dominance and additive effects, respectively. Black square and circle represent epistatic QTLs without individual effect, while interacting loci are shown by red colored bar.

In total, 6, 3, and 0 digenic interactions were detected in the E1, E2 and across-environment QTL analyses of datasets for the RIL population based on the cross (ICMB 841-P3 × 863B-P2). No digenic interactions were detected for Fe in E1 or for Zn in E2, and no digenic interactions were detected during across environment in PlabQTL (**Table 7**). However, by using the same across-environment dataset, QTLNetwork detected single digenic significant interactions with no significant main effects for Fe (*R*^2^= 5%) (**Figure 3**). None of the putative epistatic effects detected by QTLNetwork were involved in QTL × QTL × Environment (QQE) interactions.

## Discussion

Bio-fortification — breeding to develop cultivars with dense levels of micronutrients, especially Fe and Zn content in edible parts of staple foods — provides a low-cost, sustainable strategy for reducing levels of micronutrient malnutrition (Bouis, 2002). Bio-fortification is a valuable and better alternative to alleviate micronutrient deficiencies. The major advantage of growing biofortified crops is that these may produce nutrient dense grains even in relatively poor soils with target nutrients just within the critical range (Kanatti et al., 2014; Valentinuzzi et al., 2015). Presently rice, wheat, maize, common bean, sorghum and pearl millet are the crops in which research toward bio-fortification to reduce micronutrient malnutrition is going on under the umbrella of HarvestPlus Challenge Program of CGIAR. These nutritionally rich cereals will improve the health, both directly by enhancing micronutrient availability and indirectly through improved agronomic performance and crop yields (Welch, 1999; Gómez-Galera et al., 2010). In addition, seedlings grown from micronutrient-dense seeds will be vigorous and healthy hence crops will be more disease resistant and stress tolerant, ultimately resulting in improved agricultural production (Welch, 1986, 1999).

The male parent of the population was developed from the *Iniadi* landrace. This study compared estimates of mineral micronutrient content of selfed vs. OP seed samples in each of the screening environments and across seasons. The average selfed grain [Fe] was higher than the selfed grain [Zn]. Similar results were also detected by Voorrips (2002). For RILs of the (ICMB 841-P3 × 863B-P2)-derived mapping population, the average [Zn] in OP seeds was similar in both environments while the average [Fe] in OP seeds was lower in summer. In contrast, mineral contents in selfed seeds were higher than those in OP seeds and the effect of seasonal variation was also greater on selfed seeds, perhaps as a result of differences in selfed seed set in the two seasons (but this was not measured). In the summer season, the mineral contents in selfed seeds were higher indicating possible effects of environment on selfed seed set, mineral distribution, and uptake. In contrast to the present results, a previous study conducted by Velu et al. (2007) in pearl millet demonstrated that the rainy season was more favorable for mineral content in seeds than summer indicating the effect of season on seed mineral content. Vreugdenhil et al. (2004) reported that nutrient contents of plant seeds depend on environment factors. Alterations in the environment or physiology of a plant can affect the accumulation of multiple elements simultaneously. Variation in mineral uptake in different environments has been described in *Arabidopsis thaliana* (Loudet et al., 2007; Ghandilyan et al., 2009a,b) and *Silene vulgaris* (Ernst et al., 2000). Moreover, the nutrient availability in the environment not only affects the nutrient concentration of the vegetative plant parts, but also of the economic parts of a plant, and different soil types also result in micronutrient content variability (Ernst, 1974; Tyler and Zohlen, 1998; Ernst et al., 2000). Sankaran et al. (2009) also detected variation in mineral contents of seed between environments and emphasized the importance of environmental factors on quantitative traits. However, for the present study, there is no information on differences in soil conditions as both screens were conducted on similar Alfisol fields and no soil tests of micronutrient content or availability were performed, so it is not possible to determine whether soil or climatic factors were responsible for the observed differences.

There have been concerns about the possibility that high grain mineral contents may result from “concentration effects” Gomez-Becerra et al. (2010) as a consequence of small seeds, reduced seed set (which could be associated with larger selfed seed size if there is significant yield component compensation), and/or low yield capacity. Being a protogynous cross-pollinated crop, in which stigmas are normally fertilized by wind-borne pollen from other sources prior to pollen shed from flowers of the same panicle, the number of selfed seed per panicle were less in RILs and their parents compared to OP seeds per panicle (not shown). The differences in mineral contents of selfed and OP seed samples could be due to differences in numbers of seed set on per panicle dilution effect (Garvin et al., 2006). However, many studies conducted to examine the correlation between numbers of fruit and mineral/nutrient partitioning that shown the fraction of assimilates allocated to the fruits actually increased with fruit load (Hurd et al., 1979; Marcelis, 1992; Heuvelink, 1997). OP seeds were developed by a mixture of selfing and outcrossing due to the action of the wind. The effect of pollen source, xenia effect, may be another possible reason for observed differences in mineral contents between selfed and OP seed samples as the genotype of embryos and the triploid endosperm is decided after pollination. Thus OP seeds could not only be more numerous (per panicle) than self-pollinated seeds but also show xenia effects on TGM and grain mineral contents.

The analysis of variance revealed that genotypic variance was highly significant in individual environments, and broad-sense operational heritabilities were generally high enough to permit effective QTL mapping. In addition, joint analysis across the two screening environments for the (ICMB 841 × 863B)-based RIL population indicated moderately high to high proportions of genetic variance for the traits studied. In this joint analysis, G × E variance values were significant but substantially lower than genetic variance values, suggesting that the observed traits were under genetic control with limited influence of genotype × environment interaction effects, which implied there was no need for G × E partitioning. None-the-less, operational heritability estimates from these joint analyses were substantially less than those of the corresponding single-environment analyses of this RIL. These findings were largely in agreement with previous pearl millet studies conducted at ICRISAT by Ali et al. (2001) and Kannan et al. (2014) which found that G × E interactions were significant for grain yield, flowering time, plant height and panicle length. Assessment of the environmental stability of micronutrients is important in a crop improvement program aimed at enhancing the nutritional quality of food crop plants (Bidinger et al., 2007). High G × E interactions for grain Fe and Zn concentrations, which affect the rank of genotypes across the environments, have been reported in various cereal crops (Oikeh et al., 2004; Morgounov et al., 2006; Oury et al., 2006; Bidinger et al., 2007; Gomez-Becerra et al., 2010).

The broad-sense operational heritability values of 62% for selfed seed Zn content and 66% for selfed seed Fe content observed in the (ICMB 841 × 863B)-based RIL population are very promising for future application in breeding programs. Hence, based on observations of RIL progeny means, G × E variances, trait heritabilities, and variation between parents suggests that the male parents of RIL population would be highly suitable to use as donors for improving the mineral micronutrient contents in elite lines.

### Correlations

The correlation between grain Fe and Zn content has been studied in several crops, with the results, by a large, showing similar trends. In both populations, Fe and Zn contents were strongly and positively associated. This may point to common molecular mechanisms controlling the uptake and metabolism of these minerals in grains or common transporters controlling the movement of these minerals within plants (Vreugdenhil et al., 2004; Ghandilyan et al., 2006). Co-segregation of QTLs for traits might be the reason of strong association between the grain Fe and Zn content in the RIL population. The direction and intensity of association in the RIL population suggest there are good opportunities for simultaneous genetic improvement of both micronutrients (Velu et al., 2008) by co-transferring superior alleles controlling these traits into the genetic backgrounds of elite lines.

### Segregation Distortion

Distorted segregation in RIL populations derived via single-seed descent (SSD) represents the cumulative effect of both genetic and environment factors on multiple generations, and G × E interactions become more pronounced with the progress of selfing (Wang et al., 2003; Xu, 2010). In the present study, a big portion of the distorted markers was skewed toward female parent alleles. Groups of these closely linked skewed loci together make a segregation distortion region (SDR). SDRs have been reported in many crops in previous studies (Xu, 2010). Similarly, many researchers observed segregation distortion in favor of the female parent alleles (Singh and Ghai, 2007), however, segregation distortion favoring alleles from a male parent has been reported previously in pearl millet (Liu et al., 1994; Azhaguvel, 2001; Kolesnikova, 2001; Gulia, 2004; Supriya, 2010). Markers accommodated by LG1 were skewed toward male parent alleles (863B) in the (ICMB 841 × 863B)-based RIL population, while the greatest portion of distorted markers on LG2 was skewed toward female parent (ICMB 841B) alleles. In addition to genetic backgrounds, population sizes can also play and important role in the distortion phenomenon (Xian-Liang et al., 2006). Favoring of marker alleles toward one parent seems to be common and not unexpected in nature. In male gametes, pollen killers or pollen abortion, more frequently results in marker distortion as compared to disturbances in female gametes (Taylor and Ingvarsson, 2003). It has been suggested that the protogynous nature of pearl millet also contributes to segregation distortion (Liu et al., 1994; Supriya, 2010) also observed similar results in two pearl millet RIL populations genotyped with SSR and DArT markers. Finally, residual heterozygosity may also have the reason as previously demonstrated (Livingstone et al., 1999).

### Linkage Map

The published genetic maps in pearl millet have in general been created primarily with co-dominant RFLP and SSR markers. However, the linkage maps constructed in the present investigation were generated with codominant SSRs and dominant DArTs and span 1748.7 (ICMB 841 × 863B map consisting of 305 markers). Most of the SSRs used in the present study were previously mapped and published, which made easy because of the ease of use and their reproducibility. In general, different map lengths reported previously in pearl millet were the result of variation in recombination frequencies of different population structures, numbers of mapped markers, population generation, and population size. Recently Vengadessan (2008) reported the longest SSR-based skeleton linkage map for F_2_-derived F_3_ progenies with a length of 1018.7 cM accommodating only 44 well-distributed markers. Gulia (2004) also reported a large map using SSRs with map length of 748 cM for an F_2_ population. The shortest F_2_-based map (287.7 cM) reported so far Liu et al. (1994) had 181 RFLP Markers. Using a (ICMB 841 × 863B) population (ancestral to one of the populations in the current study) Yadav et al. (2004) incorporated 91 marker loci in a population of F_2_ individuals, with a map length of 617.4 cM. The same F_2_ population was used by Senthilvel et al. (2008) to obtain a map length of 677 cM with 112 loci. Using the same population but in the F_6_ generation, the present map spans 1748.7 cM. This can be explained, by the presence of a very high number of markers and small size of population (106 RILs), and a more advanced generation (expected to have twice the recombination and therefore map length as the corresponding F_2_ population). Of the 144 RILs available, only 106 could be used in present study RIL, as the remainder were excluded from the investigation because of detection of non-parental alleles (a result of outcrossing during the inbreeding generations), with a consequent reduction in population size. Several reasons can be attributed to the presence of non-parental alleles, with outcross contamination of RILs during generation advance being the most likely. The map length appears to indicate that good genome coverage has been achieved in the present study using SSRs and DArTs and that addition of more molecular markers would not serve to substantially increase the map length. As compared to previous maps in pearl millet where several regions were had marker intervals more than 50 cM, the present (ICMB 841 × 863B)-based map contains very few big gaps due to the filling of previously reported gaps leading to increment in total map length. Even with good numbers of markers and good genome coverage, the distribution of markers was not uniform. Instead, markers tended to cluster, especially at the distal ends of all chromosome arms. This suggests still there is limited chance to increase map length by increasing the number of markers.

Map length of LG1 for the (ICMB 841 × 863B) RILs in the present study was drastically increased by the addition of flanking markers. Compared with the SSR map of Senthilvel et al. (2008) two additional markers could be mapped after *Xctm27* while 46 markers were mapped before *Xpsmp2273*, which explains the observed increase in map length. One large gap of more than 50 cM remained in LG1 similar to the maps of Senthilvel et al. (2008) and Supriya (2010), using the (81B-P6 × ICMP 451-P8)-based RIL population).

LG2 also has shown an increment in map length over previous estimates ranging from 36.2 cM (Liu et al., 1994) to 179 cM (Yadav et al., 2004). The length of LG2 was comparable to that of reported by Yadav et al. (2004) as a later generation of the same cross (ICMB 841 × 863B) was used. The occurrence of additional meioses during generation advance explains the increase in the length of LG2. Senthilvel et al. (2008) reported 81.4 cM length of LG2 with a large gap of 48 cM for an F_2_ population of (ICMB 841 × 863B). Gulia (2004) also mapped two SSRs showed larger inter-marker distances in this linkage group.

LG3 was reported as the smallest among all linkage groups of pearl millet in all previous studies. But in the present study, LG3 was the second smallest after LG6. This indicates that marker numbers were not sufficient in previous investigations to cover the whole genome of LG3. In contrast to previous studies, segregation of almost all LG3 loci in both populations was distorted, which may explain part of the increase in LG3 length. Similar to previous studies reported by Liu et al. (1994), Gulia (2004), and Vengadessan (2008), a big inter-marker gap was also detected on LG3 of the (ICMB 841 × 863B)-based population in the present study.

In present study, the length of LG4 of (ICMB 841 × 863B)-based RILs was comparable with previous maps generated by Devos et al. (2000), Kolesnikova (2001), and Gulia (2004) without any large inter-marker gap like those previously reported by Liu et al. (1994) and Supriya (2010) for RILs of cross 81B-P6 × ICMP 451-P8) including above researchers. This indicates that length of LG4 of the (ICMB 841 × 863B)-based RILs will not likely increase by much with additional markers as it has good coverage with a high number of markers.

The lengths of LG5 for both populations in the present study were comparable and higher than previously reported lengths for this linkage group. The span is in agreement with that of the previously mapped F_2_ population of the cross (ICMB 841 × 863B) that showed a length of 103 cM, largest among all pearl millet populations mapped to that time Yadav et al. (2004).

LG6 of the RILs from the cross (ICMB 841 × 863B) was split into three segments in which the major segment (LG6B) accommodated 32 markers across a length of 94.2 cM. Using the F_2_ from this same population, Senthilvel et al. (2008) also detected these two big inter-marker spaces in LG6. Interestingly, the cumulative length of LG6A and LG6B is comparable with the length reported by Yadav et al. (2004) using the same F_2_ population. The consensus map generated by Qi et al. (2001) also showed that most of the loci are mapped in the center of this LG with big inter-marker spaces at distal ends and these gaps explain the segmentation of LG6 into three pieces in the present study. This indicates the there is a need to map more markers in this LG to better fill these gaps and link the three segments.

LG7 of the (ICMB 841 × 863B)-based RILs was found to be the second largest linkage group (294 cM), which is in agreement with Yadav et al. (2004). In contrast to this Gulia (2004) reported it as the pearl millet largest linkage group (195 cM). Using am (ICMB 841 × 863B)-based mapping population, Senthilvel et al. (2008) positioned marker *Xicmp3092* at the top end in LG7, while in the present study it is in position second whereas *Xctm8* was the last marker on the other end in both maps, which indicates that these markers may be in telomeric regions. There is clear cut indication of an increase in length because additional markers have been added to this linkage group compared to the earlier published maps in pearl millet. There were many large inter-marker distances observed in previous maps of this linkage group (Gulia, 2004; Yadav et al., 2004; Senthilvel et al., 2008). However, in the present map, only two big inter-marker gaps (<29 cM) were detected, which were also found in previous maps.

All linkage groups revealed a common feature of scanty marker loci and large gaps in distal ends of all linkage groups. This is likely due to hot spots of recombination in the gene-rich distal/telomeric regions of chromosomes (Devos et al., 2000; Qi et al., 2001; Senthilvel et al., 2008). The number of large gaps has decreased in the present study compared to previous linkage maps, although still there is an opportunity to map more markers to reduce or eradicate these gaps. However, (Qi et al., 2001) hypothesized that the large gaps in distal regions of pearl millet linkage groups represent the high recombination regions, rather than insufficiency of markers in these regions.

In the current study, clustering of DArTs in distal as well as central regions was more frequent than that of SSRs (Supriya et al., 2011). It seems that DArT markers may have a stronger tendency than genomic SSR and AFLP markers, in particular, to map to such gene-rich regions (Vuylsteke et al., 1999). In the present study, this occurrence may be due to usage of the methylation-sensitive restriction enzyme *Pst*I during library construction for the DArT array, and subsequent preparation of DNA samples for hybridization to the array. DArT marker clustering in distal regions of chromosome arms was observed in previous DArT mapping studies on wheat (Akbari et al., 2006; Semagn et al., 2006), barley (Wenzl and Carling, 2004), and pearl millet (Supriya et al., 2011).

### Mapping Quantitative Trait Loci (QTLs)

#### QTLs for Self-Pollinated Grain Fe Content

Although six putative QTLs were detected for this trait across individual screening environments for the (ICMB 841 × 863B)-based RILs, only one major on LG3 was consistently detected across the individual screening environment analyses and the two across-environment analyses. This suggests that the remaining variation may be the cumulative effect of many different genomic regions contributing small percentages of the phenotypic variation, with the small size of this population preventing their detection in the present study. This suggests that the transport and accumulation of minerals in seeds is a complex trait and highly influenced by environment as significant QEI was detected for self-pollinated grain Fe content in the present study. The occurrence of several QTLs for grain Fe content was also detected in various previous studies in different crops like rice (Peleg and Cakmak, 2009), *Medicago* (Sankaran et al., 2009) and wheat (Lu et al., 2008). No common QTL for Fe content in OP seeds was detected in analyses of the three data sets making all putative QTLs detected with this population for this trait environment-specific. Similarly, no common loci were observed for Fe content in self-pollinated and OP seeds of this population. This indicates that environment and pollen source play important roles on Fe content in seeds consequently different positions of detected QTLs.

#### QTLs for Self-Pollinated Grain Zn Content

In the present study, the positions of a putative major QTL on LG3 influencing the Zn content in self-pollinated as well as OP seeds were same as that detected for Fe in self-pollinated seeds. This co-localization of QTLs explains the positive correlations between contents of these two minerals. Co-localization of QTLs affecting different traits suggests either a single pleiotropic locus is involved in controlling of multiple traits or that several separate loci affecting independent traits are in close proximity (Ding and Yang, 2010). The same QTL position for Zn in self-pollinated and OP seeds also suggests that probably this QTL is less affected by variation in seed set or pollen source. Similar to Fe, a number of environments specific QTL was detected for Zn content. For example, against the five QTLs detected for Zn in OP seeds for this RIL population in the E2 screen, no QTL was detected for this trait in E1. Failure to detect QTL associated was previously reported for RIL populations of tomato (Saliba-Colombani et al., 2001), tobacco (Julio et al., 2005), and Chinese cabbage (Wu et al., 2008).

#### Co-mapped QTLs for Grain Fe and Zn Content

Quantitative traits affected by pleiotropism and linkage tend to exhibit correlations among them. This, in turn, often leads to detection of co-mapped QTLs. However, it is not easy to differentiate between linkage and pleiotropy until the QTN (Quantitative Trait Nucleotide) responsible for the phenotypic variation of each trait has been identified (Mackay, 2001). In the present study, QTLs for Fe and Zn content in the (ICMB 841 × 863B)-based RIL population were co-mapped on LG3 and favorable alleles for these QTLs were contributed by 863B (**Tables 5** and **6**). The co-localization of QTLs for contents of multiple elements was previously observed in wheat (Wu et al., 2008; Chatzav and Peleg, 2010) *Brassica oleracea* (Broadley and Hammond, 2008), *Arabidopsis* (Vreugdenhil et al., 2004), rice (Shimizu and Guerta, 2005; Stangoulis and Huynh, 2006; Ishikawa and Abe, 2009) and *Medicago* (Sankaran et al., 2009). These results suggest that some QTL regions appear to affect multiple traits. The co-mapped QTLs demonstrate the existence of genes or gene clusters with major effects on related traits. These loci might contain a gene for a common transporter such as a ZIP gene family member, which is capable of transporting Zn in addition to Fe, or a gene controlling synthesis for nicotinamide, a metal chelator involved in Fe, Zn, Cu, and Mn homeostasis (Curie and Briat, 2003; Delhaize, 2003; Sankaran et al., 2009). The stability of grain nutrient constituents across environments as well as co-localization of QTLs is of interest for both crop breeding and commercial production. In the present study, several QTLs have been identified for both minerals using the (ICMB 841 × 863B)-based RILs, with one being identified in both years and co-mapped, thereby indicated that this QTL is robust and can be targeted for marker-assisted breeding. This study also suggested that both RIL populations can be exploited for agronomic as well as mineral traits for molecular breeding.

### Epistasis

No epistasis was detected for QTLs influencing either mineral micronutrient, which suggests that marker-assisted breeding for grain Fe and Zn content would not be adversely affected by epistatic interactions associated with the putative QTLs detected from ICMB 841 and/or 863B. Assessment of QTL × QTL and QTL × QTL × Environment interactions by two-locus analysis using QTLNetwork-2.0 for the RIL population derived from cross ICMB 841 × 863B indicated that major QTLs are not involved in interactions. The main effects of the epistatic loci did not account for any phenotypic variance, but their interactions with each other produced very small and negligible effects on phenotypic variation for the observed traits. Hence interactions can be neglected during marker-assisted breeding for the observed traits using either 863B or ICMB 841 as donor parents. Epistasis analysis of this RIL population revealed that Fe content in self-pollinated seeds is largely controlled by the single main effect QTL. Only one pair of epistatic loci was identified for this trait, and their interaction did not have a big effect on phenotypic variance. Interactions of screening environments and epistasis effects also were found to be non-significant and had no effect on phenotypic variation. One reason for the absence of epistasis in the present study could be that we investigated a population developed by intra-specific cross of two well-adapted inbreds. In this case, there should be less opportunity to disrupt co-adapted epistatic gene complexes in the parents as one might expect for the inter-specific crosses that are often employed in QTL mapping studies (Melchinger et al., 1998).

## Conclusion

Improving levels of grain Fe and Zn content in pearl millet remains one of the most important breeding objectives for the nutritional security of the poor from the nations where pearl millet is consumed. The measurable genetic variation for grain Fe and Zn content as presented in the current research was used to identify major effect stable grain Fe and Zn content QTLs across two environments on LG3. This indicates that both mineral QTLs are reliable targets for marker-assisted selection (MAS).

## Author Contributions

CTH and RKS designed research. SK, CTH, RKS, NT, GS, VR, AR, and SS performed research. All authors analyzed the data. RKS, SK, CTH, RSY, and MDM wrote the paper.

## Conflict of Interest Statement

The authors declare that the research was conducted in the absence of any commercial or financial relationships that could be construed as a potential conflict of interest.

## References

[B1] Akbari M. Wenzl P. Caig V. Carling J. Xia L. Yang Kilian A. (2006). Diversity arrays technology (DArT) for high-throughput profiling of the hexaploid wheat genome. *Theor. Appl. Genet.* 113 1409–1420. 10.1007/s00122-006-0365-4 17033786

[B2] Ali A. M. Hash C. T. Ibrahim A. E. S. Raj A. G. (2001). Population diallel of elite medium-and long-duration pearl millet composites. *Crop Sci.* 41 705–711. 10.2135/cropsci2001.413705x

[B3] Allouis S. Qi X. Lindup S. Gale M. D. Devos K. M. (2001). Construction of a BAC library of pearl millet, [*Pennisetum glaucum* (L.) R. Br.]. *Theor. Appl. Genet.* 102 1200–1205. 10.1007/s001220100559

[B4] Azhaguvel P. (2001). *Linkage Map Construction and Identification of QTLs for Downy Mildew (Sclerospora graminicola) Resistance in Pearl Millet (Pennisetum glaucum* (L.) R. Br.). Madurai: Tamil Nadu Agricultural University.

[B5] Bidinger F. R. Nepolean T. Hash C. T. Yadav R. S. Howarth C. J. (2007). Identification of QTLs grain yield of pearl millet [*Pennisetum glaucum* (L.) R. Br.] in environments with variable moisture during grain filling. *Crop Sci.* 47 969–980. 10.2135/cropsci2006.07.0465

[B6] Bohra A. Jha U. C. Kumar S. (2016). “Enriching nutrient density in staple crops using modern “-Omics” tools,” in *Biofortification of Food Crops*, eds Singh U. Praharaj C. S. Singh S. S. Singh N. P. (New Delhi: Springer), 85–103.

[B7] Bouis H. E. (2002). Plant breeding: a new tool for fighting micronutrient malnutrition. *J. Nutr.* 132 491S–494S. 11880577 10.1093/jn/132.3.491S

[B8] Broadley M. R. Hammond J. P. (2008). Shoot calcium and magnesium concentrations differ between subtaxa, are highly heritable, and associate with potentially pleiotropic loci in *Brassica oleracea*. *Plant Physiol.* 146 1707–1720. 10.1104/pp.107.114645 18281414 PMC2287345

[B9] Budak H. Pedraza F. Cregan P. B. Baenziger P. S. Dweikat I. (2003). Development and utilization of SSRs to estimate the degree of genetic relationships in a collection of pearl millet germplasm. *Crop Sci.* 43 2284–2290. 10.2135/cropsci2003.2284

[B10] Cakmak I. (2008). Enrichment of cereal grains with zinc: agronomic or genetic biofortification. *Plant Soil* 302 1–17. 10.1007/s11104-007-9466-3

[B11] Cakmak I. Torun A. Millet E. Feldman M. Fahima T. Korol A. (2004). Triticum dicoccoides: an important genetic resource for increasing zinc and iron concentration in modern cultivated wheat. *Soil Sci. Plant Nutr.* 50 1047–1054. 10.1080/00380768.2004.10408573

[B12] Chatzav M. Peleg Z. (2010). Genetic diversity for grain nutrients in wild emmer wheat: potential for wheat improvement. *Ann. Bot.* 105 1211–1220. 10.1093/aob/mcq024 20202969 PMC2887062

[B13] Curie C. Briat J. F. (2003). Iron transport and signaling in plants. *Annu. Rev. Plant Biol.* 54 183–206. 10.1146/annurev.arplant.54.031902.135018 14509968

[B14] Delhaize E. (2003). Genes encoding proteins of the cation diffusion facilitator family that confer manganese tolerance. *Plant Cell* 15 1131–1142. 10.1105/tpc.009134 12724539 PMC153721

[B15] Devos K. M. Pittaway T. S. Reynolds A. Gale M. D. (2000). Comparative mapping reveals a complex relationship between the pearl millet genome and those of foxtail millet and rice. *Theor. Appl. Genet.* 100 190–198. 10.1007/s001220050026

[B16] Ding G. Yang M. (2010). Quantitative trait loci affecting seed mineral concentrations in *Brassica napus* grown with contrasting phosphorus supplies. *Ann. Bot.* 105 1221–1234. 10.1093/aob/mcq050 20237116 PMC2887070

[B17] Ernst W. H. Nelissen H. J. Ten Bookum W. M. (2000). Combination toxicology of metal-enriched soils: physiological responses of a Zn-and Cd-resistant ecotype of *Silene vulgaris* on polymetallic soils. *Environ. Exp. Bot.* 43 55–71. 10.1016/S0098-8472(99)00048-9

[B18] Ernst W. H. O. (1974). *Schwermetallvegetation der Erde.* Stuttgart: G. Fischer Verlag.

[B19] Falconer D. S. (1989). *Introduction to Quantitative Genetics*, 3rd Edn. New York: John Wiley & Sons.

[B20] Garvin D. F. Welch R. M. Finlay J. W. (2006). Historical shifts in the seed mineral micronutrient concentration of U.S. hard red winter wheat germplasm. *J. Sci. Food Agric.* 86 2213–2220. 10.1002/jsfa.2601

[B21] Ghandilyan A. Barboza L. Tisné S. Granier C. Reymond M. Koornneef M. (2009a). Genetic analysis identifies quantitative trait loci controlling rosette mineral concentrations in *Arabidopsis thaliana* under drought. *New Phytol.* 184 180–192. 10.1111/j.1469-8137.2009.02953.x 19656307

[B22] Ghandilyan A. Ilk N. Hanhart C. Mbengue M. Barboza L. Schat H. (2009b). A strong effect of growth medium and organ type on the identification of QTLs for phytate and mineral concentrations in three *Arabidopsis thaliana* RIL populations. *J. Exp. Bot.* 60 1409–1425. 10.1093/jxb/erp084 19346258

[B23] Ghandilyan A. Vreugdenhil D. Mark G. M. Aart M. (2006). Progress in the genetic understanding of plant iron and zinc nutrition. *Physiol. Plant.* 126 407–417. 10.1111/j.1399-3054.2006.00646.x

[B24] Gibson R. S. (1994). Zinc nutrition in developing countries. *Nutr. Res. Rev.* 7 151–173. 10.1079/NRR19940010 19094296

[B25] Gomez-Becerra H. F. Yazici A. Ozturk L. Budak H. Peleg Z. Morgounov A. (2010). Genetic variation and environmental stability of grain mineral nutrient concentrations in *Triticum dicoccoides* under five environments. *Euphytica* 171 39–52. 10.1007/s10681-009-9987-3

[B26] Gómez-Galera S. Rojas E. Sudhakar D. Zhu C. Pelacho A. M. Capell T. (2010). Critical evaluation of strategies for mineral fortification of staple food crops. *Transgenic Res.* 19 165–180. 10.1007/s11248-009-9311-y 19685153

[B27] Govila O. P. Madhusudhana R. Unnikrishnan K. V. (1997). Diversity in performance per se and heterosis among A1 cytoplasm pearl millet male-sterile lines. *Int. Sorghum Millets Newslett.* 38 116–119.

[B28] Graham R. D. Welch R. M. (1996). “Breeding for staple-food crops with high micronutrient density. *Paper Persented at the* *Agricultural Strategies for Micronutrients*, Washington, DC: International Food Policy Research Institute, 1–72.

[B29] Graham R. D. Welch R. M. Saunders D. A. Ortiz-Monasterio I. Bouis H. E. Bonierbale M. (2007). Nutritious subsistence food systems. *Adv. Agron.* 92 1–74. 10.2134/agronj2005.0222

[B30] Grusak M. A. Cakmak I. (2005). “Methods to improve the crop-delivery of minerals to humans and livestock,” in *Plant Nutritional Genomics*, eds Broadley M. R. White P. J. (Boca Raton, FL: CRC Press).

[B31] Gulia S. K. (2004). *QTL Mapping for Improvement of Downy Mildew [Sclerospora Graminicola (Sacc.) J. Schroet.] Resistance (DMR) in Pearl Millet [Pennisetum Glaucum (L.) R. Br.] Hybrid Parental Line ICMB* 89111. Doctoral dissertation, Chaudhary Charan Singh Haryana Agricultural University, Hisar.

[B32] Heuvelink E. (1997). Effect of fruit load on dry matter partitioning in tomato. *Sci. Hortic.* 69 51–59. 10.1016/S0304-4238(96)00993-4

[B33] Hurd R. G. Gay A. P. Mountifield A. C. (1979). The effect of partial flower removal on the relation between root, shoot and fruit growth in the indeterminate tomato. *Ann. Appl. Biol.* 93 77–89. 10.1111/j.1744-7348.1979.tb04729.x

[B34] Ishikawa S. Abe T. (2009). A major quantitative trait locus for increasing cadmium-specific concentration in rice grain is located on the short arm of chromosome 7. *J. Exp. Bot.* 61 923–934. 10.1093/jxb/erp360 20022924 PMC2814118

[B35] Julio E. Verrier J. L. Dorlhac de Borne F. (2005). Development of SCAR markers linked to three disease resistances based on AFLP within *Nicotiana tabacum* L. *Theor. Appl. Genet.* 112 335–346. 10.1007/s00122-005-0132-y 16283232

[B36] Kanatti A. Rai K. N. Radhika K. Govindaraj M. Sahrawat K. L. Rao A. S. (2014). Grain iron and zinc density in pearl millet: combining ability, heterosis and association with grain yield and grain size. *SpringerPlus* 3:763 10.1186/2193-1801-3-763 PMC4320223 25674488

[B37] Kannan B. Senapathy S. Bhasker Raj A. G. Chandra S. Muthiah A. Dhanapal A. P. (2014). Association analysis of SSR markers with phenology, grain, and stover-yield related traits in Pearl Millet (*Pennisetum glaucum* (L.) R. Br.). *Sci. World J.* 2014:562327 10.1155/2014/562327 PMC3910278 24526909

[B38] Kolesnikova M. A. (2001). *Mapping New Quantitative Trait Loci (QTL) for Downy Mildew Resistance in Pearl Millet*. Ph.D. thesis, Russian National Academy of Sciences, Moscow.

[B39] Lander E. S. Green P. Abrahamson J. Barlow A. Daly M. J. Lincoln S. E. (1987). Mapmaker: an interactive computer package for constructing primary genetic linkage maps of experimental and natural populations. *Genomics* 1 174–181. 10.1016/0888-7543(87)90010-3 3692487

[B40] Liu C. J. Witcombe J. R. Pittaway T. S. Nash M. Hash C. T. Busso C. S. (1994). An RFLP-based genetic map of pearl millet (*Pennisetum glaucum*). *Theor. Appl. Genet.* 89 481–487. 10.1007/BF00225384 24177898

[B41] Livingstone K. D. Lackney V. K. Blauth J. R. Van Wijk R. I. K. Jahn M. K. (1999). Genome mapping in *Capsicum* and the evolution of genome structure in the Solanaceae. *Genetics* 152 1183–1202. 10388833 10.1093/genetics/152.3.1183 PMC1460652

[B42] Loudet O. Saliba-Colombani V. Camilleri C. Calenge F. Gaudon V. Koprivova A. (2007). Natural variation for sulfate content in *Arabidopsis thaliana* is highly controlled by APR2. *Nat. Genet.* 39 896–900. 10.1038/ng2050 17589509

[B43] Lu K. Li L. Zheng X. Zhang Z. Mou T. Hu Z. (2008). Quantitative trait loci controlling Cu, Ca, Zn, Mn and Fe content in rice grains. *J. Genet.* 87 305–310. 10.1007/s12041-008-0049-8 19147920

[B44] Mace E. S. Buhariwalla K. K. Buhariwalla H. K. Crouch J. H. (2003). A high-throughput DNA extraction protocol for tropical molecular breeding programs. *Plant Mol. Biol. Rep.* 21 459–460. 10.1007/BF02772596

[B45] Mackay T. F. (2001). The genetic architecture of quantitative traits. *Annu. Rev. Genet.* 35 303–339. 10.1146/annurev.genet.35.102401.090633 11700286

[B46] Marcelis L. F. M. (1992). The dynamics of growth and dry matter distribution in cucumber. *Ann. Bot.* 69 487–492. 10.1093/aob/mcr150

[B47] Melchinger A. E. Utz H. F. Schön C. C. (1998). Quantitative trait locus (QTL) mapping using different testers and independent population samples in maize reveals low power of QTL detection and large bias in estimates of QTL effects. *Genet.* 149 383–403. 10.1093/genetics/149.1.383 PMC1460144 9584111

[B48] Morgounov A. Gómez-Becerra H. F. Abugalieva A. Dzhunusova M. Yessimbekova M. Muminjanov H. (2006). Iron and zinc grain density in common wheat grown in Central Asia. *Euphytica* 155 193–203. 10.1007/s10681-006-9321-2

[B49] Oikeh S. O. Menkir A. Maziya-Dixon B. Welch R. M. Glahn R. P. Gauch G. Jr. (2004). Environmental stability of iron and zinc concentrations in grain of elite early-maturing tropical maize genotypes grown under field conditions. *J. Agric. Sci.* 142 543–551. 10.1017/S0021859604004733

[B50] Oury F. X. Leenhardt F. Duperrier B. Balfourier F. Charmet G. (2006). Genetic variability and stability of grain magnesium, zinc and iron concentrations in bread wheat. *Eur. J. Agron.* 25 177–185. 10.1016/j.eja.2006.04.011

[B51] Patterson H. D. Thompson R. (1971). Recovery of inter-block information when block sizes are unequal. *Biometrika* 58 545–554. 10.1093/biomet/58.3.545

[B52] Peleg Z. Cakmak I. (2009). Quantitative trait loci conferring grain mineral nutrient concentrations in durum wheat × wild emmer wheat RIL population. *Theor. Appl. Genet.* 119 353–369. 10.1007/s00122-009-1044-z 19407982

[B53] Pfeiffer W. H. McClafferty B. (2007). HarvestPlus: breeding crops for better nutrition. *Crop Sci.* 47(Suppl. 3), S88–S105. 10.2135/cropsci2007.09.0020IPBS

[B54] Qi X. Lindup S. Pittaway T. S. Allouis S. Gale M. D. Devos K. M. (2001). Development of simple sequence repeat markers from bacterial artificial chromosomes without subcloning. *Biotechniques* 31 355–358. 11515373 10.2144/01312st08

[B55] Qi X. Pittaway T. S. Lindup S. Liu H. Waterman E. Padi F. K. (2004). An integrated genetic map and a new set of simple sequence repeat markers for pearl millet, [*Pennisetum glaucum* (L.) R. Br.]. *Theor. Appl. Genet.* 109 1485–1493. 10.1007/s00122-004-1765-y 15322756

[B56] Rai K. N. Hash C. T. Singh A. K. Velu G. (2008). Adaptation and quality traits of a germplasm-derived commercial seed parent of pearl millet. *Plant Genetic Resour. Newslett.* 154 20–24.

[B57] Rajaram V. Nepolean T. Senthilvel S. Varshney R. K. Vadez V. Rakesh K. (2013). Pearl millet [*Pennisetum glaucum* (L.) R. Br.] consensus linkage map constructed using four RIL mapping populations and newly developed EST-SSRs. *BMC Genomics* 14:159 10.1186/1471-2164-14-159 PMC3606598 23497368

[B58] Rajaram V. Varshney R. K. Vadez V. Nepolean T. Senthilvel S. Kholová J. (2010). “Development of EST resources in pearl millet and their use in development and mapping of EST-SSRs in four RIL populations,” in *Proceeding of the Plant and Animal Genome Conference* Vol. 18 San Diego, CA, 373.

[B59] Rao P. P. Birthal P. S. Reddy B. V. Rai K. N. Ramesh S. (2006). Diagnostics of sorghum and pearl millet grains-based nutrition in India. *Int. Sorghum Millets Newslett.* 47 93–96.

[B60] Robinson H. F. Comstock R. E. Harvey P. H. (1949). Estimates of heritability and degree of dominance in corn. *Agron. J.* 41 353–359. 10.2134/agronj1949.00021962004100080005x

[B61] Saliba-Colombani V. Causse M. Langlois D. Philouze J. Buret M. (2001). Genetic analysis of organoleptic quality in fresh market tomato. 1. Mapping QTLs for physical and chemical traits. *Theor. Appl. Genet.* 102 259–272. 10.1007/s001220051643

[B62] Sandstead H. H. (1991). Zinc deficiency: a public health problem? *Am. J. Dis. Child.* 145 853–859. 10.1001/archpedi.1991.02160080029016 1858720

[B63] Sankaran R. P. Huguet T. Grusak M. A. (2009). Identification of QTL affecting seed mineral concentrations and content in the model legume Medicago truncatula. *Theor. Appl. Genet.* 119 241–253. 19396421 10.1007/s00122-009-1033-2

[B64] SAS Institute Inc. (1999). *SAS/STAT User’s Guide: Version 8*. Cary, NC: SAS Institute Inc, 2.

[B65] Sehgal A. Telang S. Passah S. M. Jyothi M. C. (2004). Maternal and neonatal profile and immediate outcome in extremely low birth weight babies in Delhi. *Trop. Doct.* 34 165–168. 15267050 10.1177/004947550403400315

[B66] Semagn K. Bjørnstad Å Skinnes H. Marøy A. G. Tarkegne Y. William M. (2006). Distribution of DArT, AFLP, and SSR markers in a genetic linkage map of a doubled-haploid hexaploid wheat population. *Genome* 49 545–555. 10.1139/G06-002 16767179

[B67] Senthilvel S. Jayashree B. Mahalakshmi V. Kumar P. S. Nakka S. Nepolean T. (2008). Development and mapping of simple sequence repeat markers for pearl millet from data mining of expressed sequence tags. *BMC Plant Biol.* 8:119 10.1186/1471-2229-8-119 PMC2632669 19038016

[B68] Sharma M. Charak K. S. Ramanaiah T. V. (2003). Agricultural biotechnology research in India: status and policies. *Curr. Sci.* 84 297–302.

[B69] Shimizu A. Guerta C. Q. (2005). QTLs for nutritional contents of rice seedlings (*Oryza sativa* L.) in solution cultures and its implication to tolerance to iron-toxicity. *Plant Soil* 275 57–66. 10.1007/s11104-004-4683-5

[B70] Singh F. Nainawatee H. S. Khairwal I. S. Rai K. N. Andrews D. J. Harinarayana G. (1999). “Grain quality traits,” in *Pearl Millet Breeding*, eds Khairwal I. S. Rai K. N. Andrews D. J. Harinarayana G. (New Delhi: Vijay Primlani).

[B71] Singh K. Ghai M. (2007). An integrated molecular linkage map of diploid wheat based on a *Triticum boeoticum* × *T. monococcum* RIL population. *Theor. Appl. Genet.* 115 301–312. 10.1007/s00122-007-0543-z 17565482

[B72] Singh S. D. (1990). Sources of resistance to downy mildew and rust in pearl millet. *Plant Dis.* 74 871–874. 10.1094/PD-74-0871

[B73] Stangoulis J. C. R. Huynh B. L. (2006). Quantitative trait loci for phytate in rice grain and their relationship with grain micronutrient content. *Euphytica* 154 289–294. 10.1007/s10681-006-9211-7

[B74] Supriya A. (2010). *Genetic Diversity Analysis and QTL Mapping in Pearl Millet (Pennisetum glaucum) Using Diversity Arrays Technology (DArT)*. Doctoral dissertation, Chaudhary Charan Singh Haryana Agricultural University.

[B75] Supriya A. Senthilvel S. Nepolean T. Eshwar K. Rajaram V. Shaw R. (2011). Development of a molecular linkage map of pearl millet integrating DArT and SSR markers. *Theor. Appl. Genet.* 123 239–250. 10.1007/s00122-011-1580-1 21476042

[B76] Taylor D. R. Ingvarsson P. K. (2003). Common features of segregation distortion in plants and animals. *Genetica* 117 27–35. 10.1023/A:1022308414864 12656570

[B77] Tyler G. Zohlen A. (1998). Plant seeds as mineral nutrient resource for seedlings—a comparison of plants from calcareous and silicate soils. *Ann. Bot.* 81 455–459. 10.1006/anbo.1997.0581

[B78] Utz H. F. Melchinger A. E. (1996). PLABQTL: a program for composite interval mapping of QTL. *J. Quant. Trait Loci* 2 1–5. 10.1094/PHYTO.2004.94.8.862

[B79] Valentinuzzi F. Mason M. Scampicchio M. Andreotti C. Cesco S. Mimmo T. (2015). Enhancement of the bioactive compound content in strawberry fruits grown under iron and phosphorus deficiency. *J. Sci. Food Agric.* 95 2088–2094. 10.1002/jsfa.6924 25244604

[B80] Van Os H. Stam P. Visser R. G. Van Eck H. J. (2005). RECORD: a novel method for ordering loci on a genetic linkage map. *Theor. Appl. Genet.* 112 30–40. 10.1007/s00122-005-0097-x 16228189

[B81] Velu G. Kulkarni V. N. Muralidharan V. Rai K. N. Longvah T. Sahrawat K. L. (2006). A rapid screening method for grain iron content in pearl millet. *Internat. Sorghum Millets Newslett.* 47 158–161.

[B82] Velu G. Rai K. N. Muralidharan V. Kulkarni V. N. Longvah T. Raveendran T. S. (2007). Prospects of breeding biofortified pearl millet with high grain iron and zinc content. *Plant Breed.* 126 182–185. 10.1111/j.1439-0523.2007.01322.x

[B83] Velu G. Rai K. N. Sahrawat K. L. (2008). Variability for grain iron and zinc content in a diverse range of pearl millet populations. *J. Crop Improve.* 35 186–191.

[B84] Vengadessan V. (2008). *Genetic and QTL Analyses of Sink Size Traits in Pearl Millet (Pennisetum glaucum (L.) R. Br.)*. Doctoral dissertation, Tamil Nadu Agricultural University, Coimbatore.

[B85] Voorrips R. E. (2002). MapChart: software for the graphical presentation of linkage maps and QTLs. *J. Heredity* 93 77–78. 10.1093/jhered/93.1.77 12011185

[B86] Vreugdenhil D. Aarts M. G. M. Koornneef M. Nelissen H. Ernst W. H. O. (2004). Natural variation and QTL analysis for cationic mineral content in seeds of *Arabidopsis thaliana*. *Plant Cell Environ.* 27 828–839. 10.1111/j.1365-3040.2004.01189.x

[B87] Vuylsteke M. Mank R. Antonise R. Bastiaans E. Senior M. L. Stuber C. W. (1999). Two high-density AFLP^®^ linkage maps of *Zea mays* L.: analysis of distribution of AFLP markers. *Theor. Appl. Genet.* 99 921–935. 10.1007/s001220051399

[B88] Wang J. Van Ginkel M. Podlich D. Ye G. Trethowan R. Pfeiffer W. (2003). Comparison of two breeding strategies by computer simulation. *Crop Sci.* 43 1764–1773. 10.1017/S1751731112000341

[B89] Welch R. M. (1986). Effects of nutrient deficiencies on seed production and quality. *Adv. Plant Nutr.* 2 205–247.

[B90] Welch R. M. (1999). “Importance of seed mineral nutrient reserves in crop growth and development,” in *Mineral Nutrition of Crops. Fundamental Mechanisms and Implications*, ed.Rengel Z. (New York: Food Products Press), 205–226.

[B91] Welch R. M. Graham R. D. (2002). Breeding crops for enhanced micronutrient content. *Plant Soil* 245 205–214. 10.1023/A:1020668100330

[B92] Wenzl P. Carling J. (2004). Diversity arrays technology (DArT) for whole-genome profiling of barley. *Proc. Natl. Acad. Sci. U.S.A.* 101 9915–9920. 10.1073/pnas.0401076101 15192146 PMC470773

[B93] White P. J. Broadley M. R. (2009). Biofortification of crops with seven mineral elements often lacking in human diets–iron, zinc, copper, calcium, magnesium, selenium and iodine. *New Phytol.* 182 49–84. 10.1111/j.1469-8137.2008.02738.x 19192191

[B94] Wu J. Yuan Y. X. Zhang X. W. Zhao J. Song X. Li Y. (2008). Mapping QTLs for mineral accumulation and shoot dry biomass under different Zn nutritional conditions in Chinese cabbage (*Brassica rapa* L. ssp. pekinensis). *Plant Soil* 310 25–40. 10.1007/s11104-008-9625-1

[B95] Xian-Liang S. Xue-Zhen S. Tian-Zhen Z. (2006). Segregation distortion and its effect on genetic mapping in plants. *Chin. J. Agric. Biotechnol.* 3 163–169. 10.1079/CJB2006110

[B96] Xu Y. (2010). *Molecular Plant Breeding.* Wallingford: Cab International Publishing location.

[B97] Yadav R. S. Hash C. T. Bidinger F. R. Devos K. M. Howarth C. J. (2004). Genomic regions associated with grain yield and aspects of post-flowering drought tolerance in pearl millet across stress environments and tester background. *Euphytica* 136 265–277. 10.1023/B:EUPH.0000032711.34599.3a

[B98] Zeng Z. B. (1994). Precision mapping of quantitative trait loci. *Genetics* 136 1457–1468. 8013918 10.1093/genetics/136.4.1457 PMC1205924

